# Methylglyoxal, a Reactive Glucose Metabolite, Induces Bladder Overactivity in Addition to Inflammation in Mice

**DOI:** 10.3389/fphys.2020.00290

**Published:** 2020-04-03

**Authors:** Mariana G. de Oliveira, Matheus L. de Medeiros, Edith B. G. Tavares, Fabiola Z. Mónica, Edson Antunes

**Affiliations:** Department of Pharmacology, University of Campinas (UNICAMP), Campinas, Brazil

**Keywords:** advanced glycation end products, cystometry, transient receptor potential, muscarinic receptors, NF-kB, inducible nitric oxide synthase

## Abstract

Diabetic bladder dysfunction (DBD) is one of the most common complication of diabetes. Methylglyoxal (MGO), a highly reactive dicarbonyl compound formed as a by-product of glycolysis, is found at high levels in plasma of diabetic patients. Here, we explored the effects of chronic administration of MGO on micturition pattern (cystometry) and bladder contractility *in vitro* in healthy male C57/BL6 mice. Methylglyoxal was given at 0.5% in drinking water for 4 weeks. Exposure to MGO led to bladder tissue disorganization, edema of lamina propria, partial loss of urothelium and multiple leukocyte infiltrates. Filling cystometry revealed significant increases of micturition frequency and number of non-voiding contractions (NVCs) in the MGO group, clearly indicating an overactive bladder profile. Bladder contractions induced by electrical-field stimulation (EFS) and carbachol were significantly higher in the MGO group, while the muscarinic M_2_ and M_3_ mRNA expressions remained unchanged between groups. Additionally, MGO exposure induced upregulation of TRPA1 and down-regulation of TRPV1 and TRPV4 in bladder tissues. Methylglyoxal did not change the mRNA expression of the advanced glycation end products receptor (RAGE), but markedly increased its downstream NF-κB - iNOS signaling. The mRNA expression of cyclooxygenase-2 (COX-2) and reactive-oxygen species (ROS) levels remained unchanged. Altogether, our data show that 4-week MGO intake in mice produces an overactive bladder phenotype in addition to bladder inflammation and increased NF-kB/iNOS signaling. TRPA1 up-regulation and TRPV1/TRPV4 down-regulation may account for the MGO-induced bladder overactivity. Scavengers of MGO could be an option to ameliorate bladder dysfunction in diabetic conditions.

## Introduction

Diabetes mellitus is a chronic progressive metabolic disorder with multiple serious complications (American Diabetes Association (ADA), 2018). The negative impact of both types 1 and 2 diabetes in the lower urinary tract function has been long recognized. Diabetic bladder dysfunction (DBD) is one of the most common complications of diabetes, affecting up to 50% of the patients (Gomez et al., 2011). Taking into consideration the predictions of nearly 439 million diabetic individuals in 2030 (Shaw et al., 2010), a large proportion of them is expected to be afflicted by DBD, which although not a life-threatening condition, it greatly impairs the individual’s quality of life. The pathophysiology of DBD is multifactorial and may include alterations in the detrusor and urethra smooth muscles, urothelium, autonomic nerves and blood vessels (Yoshimura et al., 2005). Recently, DBD was recognized as a progressive group of bladder symptoms, characterized in the early stages by bladder overactivity and increased urinary frequency, while in the later stages, after prolonged hyperglycemia and insulin resistance, by an insensible and decompensated bladder, resulting in impaired bladder emptying and high residual volumes (Yuan et al., 2015; Daneshgari et al., 2017). Despite the advances in understanding DBD, the mechanisms underlying bladder dysfunction in diabetic patients are incompletely understood.

Methylglyoxal (MGO), a highly reactive dicarbonyl compound, is endogenously formed in all cell types as a by-product of glucose metabolism (Allaman et al., 2015). High levels of MGO occur when the concentrations of its precursors are elevated, for instance, during hyperglycemia (Allaman et al., 2015). Compared to healthy individuals, two- to four-fold higher levels of MGO is found in the plasma and urine diabetic patients (Odani et al., 1999; Lu et al., 2011; Senda et al., 2015; Pastor-Belda et al., 2017). MGO is one of the most potent glycating agents and is a potential precursor of advanced glycation end products (AGEs), which have been proposed as an important mechanism contributing to the pathophysiology of several complications in diabetes (Shamsaldeen et al., 2016; Hanssen et al., 2018), including diabetic nephropathy (Hanssen et al., 2019) and vascular dysfunction (Brouwers et al., 2010).

Methylglyoxal-derived AGEs may interact with selective receptors, termed RAGE, a transmembrane protein of the immunoglobulin superfamily (Eleazu et al., 2019). RAGE activation triggers a signaling cascade involving activation of the nuclear transcription factor NF-κB and pro-inflammatory pathways, including the stimulation of inducible nitric oxide synthase (iNOS) (Goldin et al., 2006; Dhar et al., 2010; Wang et al., 2019). *In vivo*, chronic MGO administration to rats causes diabetes-like microvascular changes (Berlanga et al., 2005), contributing to endothelial (Sena et al., 2012) and erectile dysfunctions (Dalaklioglu et al., 2018). In addition, MGO has been shown to activate transient receptor potential (TRP) channels, a superfamily of cation channels involved in several cellular functions, including nociception and mechanosensory transduction in the bladder (Andersson, 2019). Previous studies showed that activation of transient receptor potential ankyrin 1 (TRPA1) by MGO contributes to hyperalgesia in diabetic neuropathy (Bierhaus et al., 2012; Andersson et al., 2013).

The findings described above highlight the role of MGO in several complications of diabetes; however, little is known about its effects on the bladder. We hypothesized that MGO accumulation directly influences the bladder function, inducing a dysfunctional phenotype that resembles the early stages of DBD, and may be an additional underlying mechanism for DBD initiation and progression, independently of insulin resistance and hyperglycemia. Therefore, by chronic administration of MGO to healthy mice, we explored the effects of MGO on the micturition function by evaluating both the urodynamic profile in anesthetized mice (filling cystometry) and the *in vitro* bladder responses to both contractile agents and electrical-field stimulation (EFS). The bladder mRNA expressions of muscarinic M_2_ and M_3_ receptors, as well as TRPA1, related to bladder nociception, and of TRP vanilloid 1 (TRPV1) and vanilloid 4 (TRPV4), related to bladder filling sensation, were also evaluated. The inflammatory responses in the bladder were investigated through histological analysis and mRNA expressions of RAGE, NF-κB, iNOS and cyclooxygenase-2 (COX-2), as well as by the measurement of reactive-oxygen species (ROS) levels in the bladder tissues.

## Materials and Methods

### Animals

Sixty male C57BL/6 mice, 5 week-old, were housed in cages (*n* = 3 per cage) located in a ventilated cage shelters with constant humidity of 55 ± 5% and temperature of 24 ± 1°C under a 12 h light-dark cycle and received standard food *ad libitum*. Euthanasia was performed by overdose of isoflurane, in which animals were exposed to a concentration greater than 5% until one min after breathing stops. Cervical dislocation was performed to confirm the euthanasia. Animals studies are reported in compliance with the ARRIVE guidelines.

### Experimental Groups

Animals were randomly separated into different groups for *in vitro* and *in vivo* studies (*n* = 6 mice per group). An independent person not involved in data acquisition and analysis randomly assigned animals to the operators. The animals were assigned in two experimental groups: Control (*n* = 30) and Methylglyoxal (MGO, *n* = 30). Animals from the MGO group received 0.5% MGO (Sigma Aldrich, Missouri, United States) in the drinking water for 4 weeks, while the control group received filtered water. The optimal dose of MGO was estimated based on previous studies in rodents with modifications (Berlanga et al., 2005; Sena et al., 2012).

### Mice Weight and Glycemic Control

After 4 weeks of treatment, blood samples were collected from the tail after 8 h fasting period and taken for serum glucose determination using ACCUCHECK Blood Glucose Monitoring System^®^ (Roche Diagnostics, Indianapolis, United States). After euthanasia, total body and bladder weights, as well as the relative bladder weight (bladder to body ratio) were determined.

### Histological Analysis

Bladders were excised and fixed in 4% paraformaldehyde for 24 h, dehydrated in ethanol and embedded in paraffin. Tissues were sliced (5 μm sections) on a microtome (Leica, Wetzlar, Germany), dewaxed in xylene, rehydrated in gradient alcohol and stained with hematoxylin and eosin for light microscopy examination. Digital images were obtained with a microscope Eclipse 80i (Nikon, Tokyo, Japan) equipped a digital camera (DS-U3, Nikon).

### Cystometric Analysis

Mice were anesthetized by intraperitoneal injection of urethane (1.5 g/kg). A 1-cm abdominal incision was made to expose the bladder and a 25-gauge cannula was inserted in the bladder dome. The cannula was connected to a 3-way tap, of which 1 port was connected to the infusion pump through a PE-50 catheter. Before starting cystometry, the bladder was emptied and continuous cystometry was performed by infusing saline in the bladder at 0.6 mL/h for 45 min after the end of the first micturition cycle. The following parameters were assessed: basal pressure (minimum pressure between two micturition), capacity (volume needed to induce first micturition), voiding pressure (pressure reached during micturition), frequency of voiding and number of non-voiding contractions (NVCs, spontaneous bladder contractions higher than 4 mmHg from the baseline pressure that did not result in a void) (de Oliveira et al., 2018). One mouse was used for each cystometrogram and euthanatized immediately after the experimental protocol.

### Functional Assays in Isolated Bladder

After euthanasia, the bladders were removed and two longitudinal bladder smooth muscle strips with intact mucosa were obtained from each animal (de Oliveira et al., 2018). Tissues were mounted in organ baths filled with Krebs−Henseleit solution (117 mM NaCl, 4.7 mM KCl, 2.5 mM CaCl_2_, 1.2 mM MgSO_4_, 1.2 mM KH_2_PO_4_, 25 mM NaHCO_3_ and 11 mM Glucose, pH 7.4) continuously bubbled with a mixture of 95% O_2_ and 5% CO_2_. Resting tension was adjusted for 5 mN. Tissues were allowed to equilibrate for 1 h and changes in isometric force were recorded using a PowerLab system (ADInstruments Inc., Sydney, AU). Cumulative concentration-response curves (CRCs) to the muscarinic receptor agonist carbachol (1 nM to 100 μM) and to the depolarizing agent potassium chloride (KCl, 80 mM) were obtained in bladder strips. Non-linear regression analysis to determine the potency (pEC50) was carried out using GraphPad Prism (GraphPad Software, Inc., California, United States) with the constraint that F = 0. All concentration-response data were evaluated for a fit to a logistics function in the form: E = Emax/([1 + (10c/10x) n] + F), where E is the effect of above basal, Emax is the maximum response produced by agonists; c is the logarithm of the pEC50, the concentration of drug that produces a half maximal response; x is the logarithm of the concentration of the drug; the exponential term, n, is a curve-fitting parameter that defines the slope of the concentration–response line, and F is the response observed in the absence of added drug. The contractile responses were expressed as mN per milligram of tissue (mN/mg).

### Electrical-Field Stimulation in Isolated Bladder

In separate assays, electrical-field stimulation (EFS) was applied to bladder strips placed between two platinum ring electrodes connected to a stimulator (Grass Technologies, Rodhe Island, United States). EFS was conducted at 80 V, 1 ms pulse width and trains of stimuli lasting 10 s at varying frequencies (1–32 Hz). The contractile responses were expressed as mN/mg.

### Quantitative Real-Time RT-PCR (qPCR)

Total RNA was extracted from freshly dissected bladders using TRIzol^®^ reagent (Invitrogen, Mississippi, United States) according to the manufacturer’s protocol. DNase treated RNA samples were then transcribed with High Capacity Reverse Transcription Kit^®^ (Applied Biosystems, California, United States). cDNA samples concentrations were quantified using a spectrophotometer (Nanodrop Lite^®^, Thermo Scientific, Massachusetts, United States). Synthetic oligonucleotide primers (Table 1) were obtained from Integrated DNA Technologies (Iowa, United States) and Qiagen (Hilden, Germany). The reactions were performed with 10 ng cDNA, 6 μl SYBR Green Master Mix^®^ (Life Technologies, California, United States) and the optimal primer concentration, in a total volume of 12 μl. Real-time PCR was performed in the equipment StepOne-Plus^®^ Real Time PCR System (Applied Biosystems). The reaction program was 95°C for 10 min, followed by 40 cycles of 95°C for 15 s then 60°C for 1 min. At the end of a normal amplification, a degradation time was added, during which the temperature increased gradually from 60°C to 95°C. Threshold cycle (Ct) was defined as the point at which the fluorescence rises appreciably above the background fluorescence. Two replicas were run on the plate for each sample, and each sample was run twice independently. To determine the specificity of the amplification, the melting curve analysis of the PCR products was performed to ensure that only one fragment was amplified. The 2^–ΔΔ*Ct*^ method was utilized to analyze the results, which were expressed by the difference between Ct values of chosen genes and the housekeeping gene β–actin. The signal strength for β–actin did not differ between groups (21.08 ± 0.45 and 20.36 ± 0.16, for control and MGO, respectively, analyzed by unpaired *t*-test).

**TABLE 1:** Primer sequences used for real-time PCR amplifications.

Gene	Forward	Reverse
**IDT Integrated DNA Technologies**		
M_2_-mAChR	5′-CACGGTTTCCACTTCCCTG-3′	5′-TGCATGCGTCACCCTTTTG-3′
M_3_-mAChR	5′-CCCACAGGCAGTTCTCGAA-3′	5′-CCTCCTAGATGACCGTTTCGT-3′
NF-κB	5′-ATGGCAGACGATGATCCCTAC-3′	5′-TGTTGACAGTGGTATTTCTGGTG-3′
RAGE	5′-CTGAACTCACAGCCAGTGTCCC-3′	5′-CCCTGACTCGGAGTT-3′
TRPA1	5′-GCAGGTGGAACTTCATACCAACT-3′	5′-CACTTTGCGTAAGTACCAGAGTGG-3′
TRPV1	5′-CAGCTGACAACACCAAGTTC-3′	5′-CAGTGACACGGAAATAGTC-3′
TRPV4	5′-CGGACCACAGTGGACTACCT-3′	5′-AGCCATCGACGAAGAGAGAA-3′

**Gene**	**Catalog number**	**GenBank Accession Number**

**Qiagen Quantitec Primers Assays**		
ACTB	QT00095242	NM_007393
COX-2	QT00165347	NM_011198
iNOS	QT00100275	NM_010927

*ACTB, actin beta; COX-2, cyclooxygenase-2 2; iNOS, inducible nitric oxide synthase; M_2_-mAChR, muscarinic acetylcholine receptor M_2_; M_3_-mAChR, muscarinic acetylcholine receptor M_3_; NF-κB, nuclear factor kappa B; RAGE; advanced glycosylation end product receptor; TRPA1, transient receptor potential ankyrin 1; TRPV4, transient receptor potential vanilloid 1; TRPV4, transient receptor potential vanilloid 4.*

### Measurement of ROS Levels

The oxidative fluorescent dye dihydroethidium (DHE) was used to evaluate *in situ* ROS generation. The bladders was embedded in a freezing medium and transverse sections (12 μm) were obtained on a cryostat, collected in glass slides, equilibrated for 10 min in Hank’s solution (1.6 mM CaCl_2_, 1.0 mM MgSO_4_, 145.0 mM NaCl, 5.0 KCl, 0.5 mM NaH_2_PO_4_, 10.0 mM Glucose, 10.0 HEPES, pH 7.4). Fresh Hank’s DHE solution (2 μM) was applied to each tissue section, and the slides incubated in a light-protected humidified chamber at 37°C for 30 min. Images were obtained with a microscope (Eclipse 80i, Nikon, Tokyo, Japan) equipped for epifluorescence (excitation at 488 nm; emission at 610 nm) and a digital camera (DS-U3, Nikon). Fluorescence was detected with a 585 nm long pass filter. The number of nuclei labeled with ethidium bromide in detrusor smooth muscle and urothelium wall was automatically counted using Image J software (NIH, Maryland, United States), and expressed as labeled nuclei per millimeter squared.

### Statistical Analysis

Data were expressed as the mean ± standard error of the mean (SEM) of 6 animals per group. The group sizes referred to independent values not replicates. The software GraphPad Prism Version 6 (GraphPad Software Inc.) was used for all statistical analysis. All statistical comparisons have been pre-planned and reported irrespective of outcome, i.e., whether *p* was <0.05 or not. Unpaired Student’s *t*-test was used to assess the results. *p* < 0.05 was taken as showing a significant difference.

## Results

### General Characteristics and Bladder Histology

After 4 weeks treatment, MGO given in the drinking water resulted in a small (despite significant) reduction of body weight by about of 12% (Table 2; *p* < 0.05). No significant changes in the total or relative bladder weight were observed in MGO compared with control group (Table 2). In addition, mice from the MGO group demonstrated normoglycemia after 8 h fasting, with serum glucose levels close to the control levels (Table 2).

**TABLE 2:** General characteristics of control and methylglyoxal (MGO)-exposed mice.

	Control	MGO
Total body weight (g)	25.4 ± 2.1	22.4 ± 1.0*
Total bladder weight (mg)	29.0 ± 3.8	29.0 ± 12.3
Relative bladder weight (mg/g)	1.1 ± 0.2	1.3 ± 0.6
Serum glucose (mg/dl)	142 ± 20	156 ± 24

Values were expressed as mean ± SEM of 12 animals/group. *p < 0.05 compared with control group.

**FIGURE 1:**
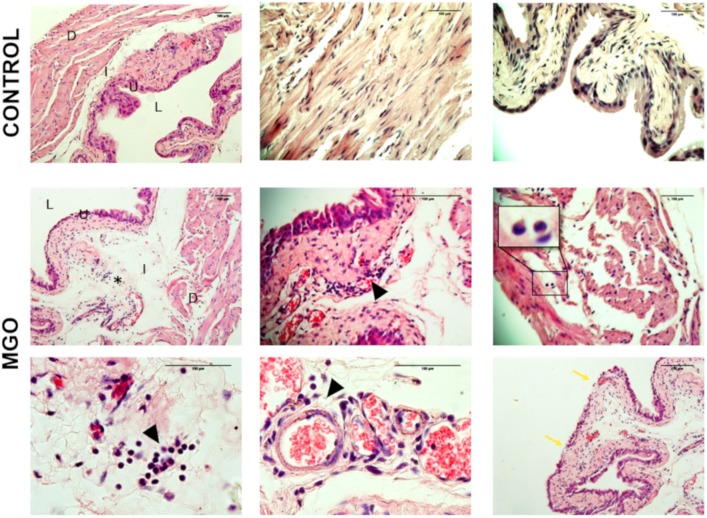
Cross-sections histological evaluation (hematoxylin and eosin staining) of bladders from control (1st row) and methylglyoxal (MGO, 2nd, and 3rd rows) exposed animals depicting edema (asterisk), urothelial damage (blue arrow), and leukocyte infiltration (black arrowhead, inset). D, detrusor smooth muscle; S, submucosa; U, urothelium; L, lumen. Scale bar 100 μm in all panels.

Histological evaluation of the bladder tissues from control animals demonstrated tissue organization with distinct compartments, including a multi-layer urothelium, the lamina propria and smooth muscle bundles (Figure 1). Neither edema nor hemorrhage was observed. In contrast, bladders from MGO-exposed mice revealed clear tissue disorganization, reduction in the number of layers of urothelial cells, pronounced mucosal edema along with eosinophil and lymphocyte infiltration (Figure 1).

### Methylglyoxal Exposure Induces Bladder Overactivity

**FIGURE 2:**
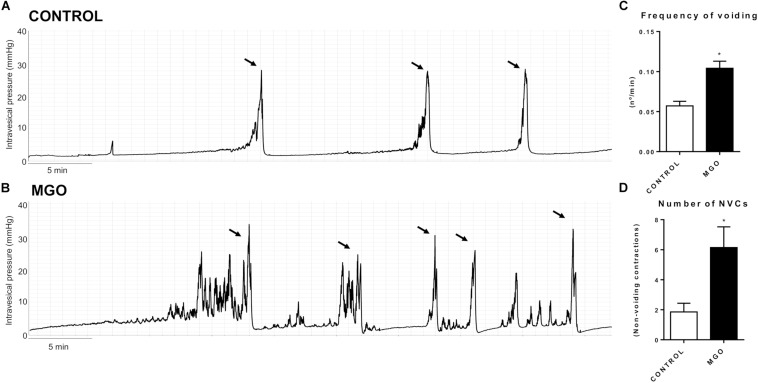
Representative traces of filling cystometry in anesthetized mice from groups control (**A**) and methylglyoxal (MGO)-exposed (**B**, 0.5% in drinking water for 4 weeks). Arrows indicate voiding. Changes in the frequency of voiding and number of non-voiding contractions (NVCs) are represented in **C** and **D**, respectively. Data are means ± SEM (*n* = 6). *p < 0.05 vs. CONTROL, unpaired *t*-test.

Cystometric recordings in anesthetized mice were carried out in both control and MGO-treated mice through intravesical infusion of saline solution. The micturition pattern in control mice was regular with rare NVCs (Figure 2A). The exposure to MGO (Figure 2B) induced an overactive bladder pattern characterized by marked increases in voiding frequency (Figure 2C), which was by about of 1.8-fold higher in MGO than the control group (*p* < 0.05), and also in number of NVCs (Figure 2D), which was by about of 3.3-fold higher in the MGO group (*p* < 0.05). No significant differences for basal, threshold, voiding and post-voiding pressures, and bladder capacity were found between control and MGO groups (Table 3).

**TABLE 3:** Cystometric parameters of anesthetized control and methylglyoxal (MGO)-exposed mice.

	Control	MGO
Basal pressure (mmHg)	3.40 ± 0.36	3.68 ± 0.80
Threshold pressure (mmHg)	6.40 ± 0.82	5.69 ± 0.98
Voiding pressure (mmHg)	33.40 ± 1.80	28.8 ± 5.87
Bladder capacity (mL)	0.17 ± 0.03	0.19 ± 0.03
Post-voiding pressure (mmHg)	2.58 ± 0.42	1.79 ± 0.48
Frequency of voiding (N^o^/min)	0.06 ± 0.01	0.10 ± 0.01*
Number of NVCs (N^o^/min)	1.85 ± 0.58	6.14 ± 1.38*

NVCs, non-voiding contractions. Values were expressed as mean ± SEM of 6 animals/group. *p < 0.05 compared with control group.

### Methylglyoxal Exposure Induces Bladder Smooth Muscle Hypercontractility *in vitro*

**FIGURE 3:**
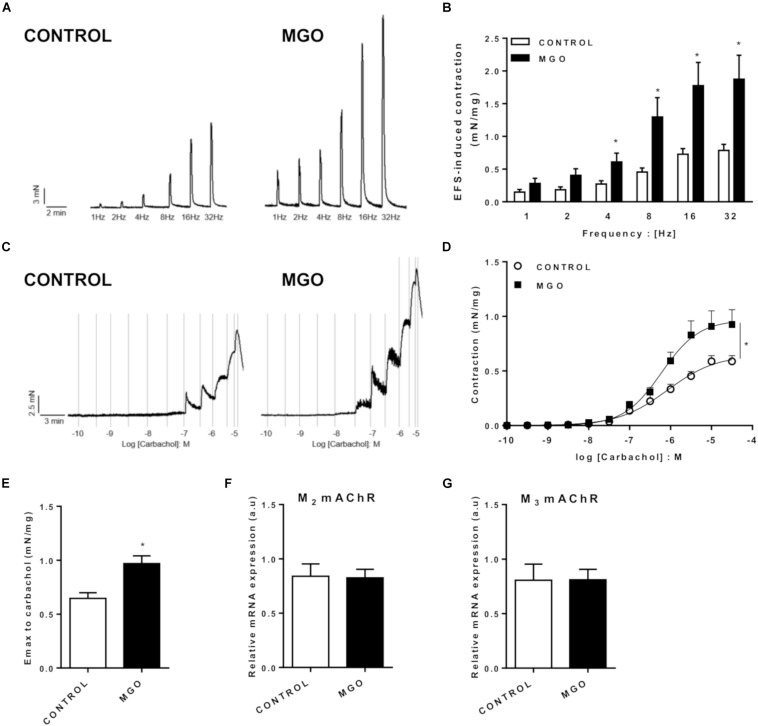
Functional *in vitro* evaluation of isolated bladders from control and methylglyoxal (MGO)- exposed mice. Electrical field stimulation (EFS)-induced bladder contraction (**A** and **B**). Concentration-response curves (**C** and **D**) and maximal response (Emax, **E**) to the muscarinic agonist carbachol. mRNA expression levels of the M_2_ (**F**) and M_3_ (**G**) muscarinic acetylcholine receptors (mAChR). The mRNA expression levels of each gene were normalized to β-actin expression levels and the values are expressed in arbitrary units (a.u). Data are means ± SEM (*n* = 6). *p < 0.05 vs. CONTROL, unpaired *t*-test.

In the bladder strips, the contractile responses elicited by EFS (1 to 32 Hz) produced frequency-dependent contractions in both control and MGO groups, but the responses were significantly higher in the MGO group at 4 to 32 Hz-stimulation (Figures 3A,B, *p* < 0.05). Furthermore, the addition of the muscarinic agonist carbachol (0.001 to 100 μM) produced concentration-dependent bladder contractions in all groups (Figures 3C,D), but the maximal contractile responses (Emax, Figure 3E) were significantly increased in MGO-exposed mice compared with the control group (*p* < 0.05). No statistically significant differences for the potency (pEC50) of carbachol were found between both groups (6.07 ± 0.15 and 6.22 ± 0.13 for control and MGO, respectively). Additionally, as shown in Figures 3F,G, qPCR analysis of isolated bladders revealed that MGO exposure did not induce significant alterations of M_2_ or M_3_ muscarinic receptors mRNA expressions. No significant differences in KCl (80 mM)-induced contractions were observed between control and MGO groups (0.43 ± 0.14 and 0.61 ± 0.12 mN/mg, respectively).

### mRNA Expressions of NF-κB, iNOS, RAGE and COX-2

**FIGURE 4:**
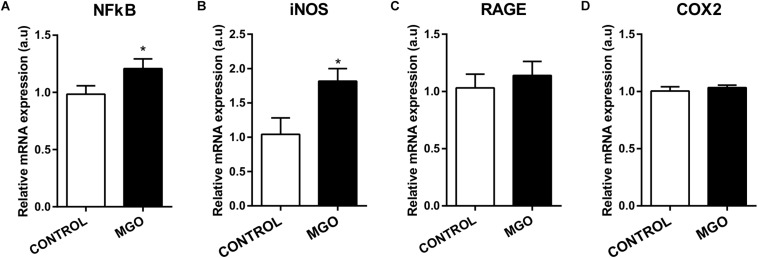
mRNA expression levels of the transcription factor NF-κB (**A**), inducible nitric oxide synthase (iNOS, **B**), advanced glycation end products receptor (RAGE, **C**) and cyclooxygenase 2 (COX2, **D**). The mRNA expression levels of each gene were normalized to β-actin expression levels and the values are expressed in arbitrary units (a.u). Data are means ± SEM (*n* = 6). *p < 0.05 vs. CONTROL, unpaired *t*-test.

qPCR Analysis of isolated bladders revealed that MGO exposure increased significantly the mRNA expression of NF-κB (Figure 4A) and iNOS (Figure 4B) when compared to the control group (*p* < 0.05). No alterations of mRNA expression for RAGE (Figure 4C) and COX-2 (Figure 4D) were observed in bladders of MGO compared with control group.

### ROS Levels

**FIGURE 5:**
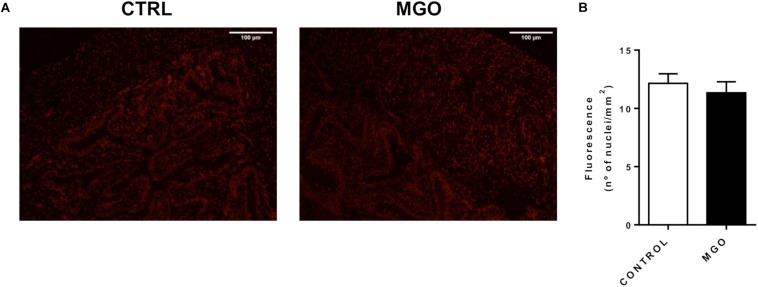
Reactive oxygen species (ROS) generation in bladders from control and methylglyoxal (MGO)- exposed mice. Representative images of ROS measurement through dye dihydroethidium-induced fluorescence in bladder sections (12 μm, **A**). Scale bar 100 μm. Quantification of ethidium bromide-positive nuclei (**B**). Data are means ± SEM (*n* = 6). Unpaired *t*-test.

The reactive-oxygen species levels in the detrusor smooth muscle and urothelium were measured by the fluorescent dye DHE in fresh frozen bladder sections (Figures 5A,B). However, quantification of the fluorescence revealed no significant changes in ROS levels were seen between groups (12.2 ± 0.8 and 11.3 ± 0.9 nuclei/mm^2^, for control and MGO groups, respectively).

### Methylglyoxal Modulates TRP Channels Expression

**FIGURE 6:**
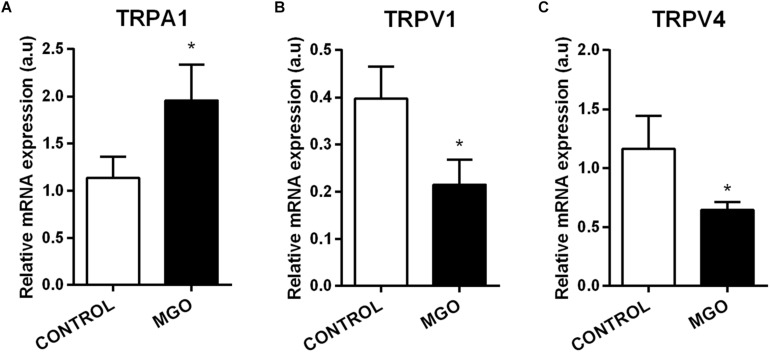
mRNA expression levels of the transient receptor potential channels sub-types ankyrin type-1 (TRPA1, **A**), vanilloid type-1 (TRPV1, **B**), and vanilloid type-4 (TRPV4, **C**). The mRNA expression levels of each gene were normalized to β-actin expression levels and the values are expressed in arbitrary units (a.u). Data are means ± SEM (*n* = 6). *p < 0.05 vs. CONTROL, unpaired *t*-test.

Quantitative PCR analysis of isolated bladders revealed that MGO exposure induces significant increase of TRPA1 mRNA expression (Figure 6A) but significant decreases of TRPV1 (Figure 6B) and TRPV4 (Figure 6C) when compared to the control groups (*p* < 0.05).

## Discussion

Methylglyoxal, a major dicarbonyl compound found in elevated concentrations in plasma of diabetic patients, has been emerged as a key player in the development of diabetic complications, including diabetic chronic kidney disease (Hanssen et al., 2019). However, no previous study examined the effects of MGO on the regulation of bladder function. In the present study, the urodynamic evaluation of MGO-exposed mice revealed significant increases of micturition frequency and number of NVCs, which clearly indicates an overactive bladder profile. These changes have consistently been shown by other investigators in different animal models of diabetes and obesity (Leiria et al., 2011; He et al., 2016; Calmasini et al., 2017). The increased NVCs are mirrored by the increased contractile activity *in vitro* for carbachol and EFS, which agrees with previous studies in rats and mice (Kendig et al., 2016; de Oliveira et al., 2018). Muscarinic receptors in the detrusor smooth muscle are physiologically the major mechanism to elicit urinary bladder contraction (Andersson and Arner, 2004). The direct activation of muscarinic receptors by carbachol produced significantly higher contractions in bladders from MGO compared with control group with no concomitant changes in muscarinic M_2_ and M_3_ receptor mRNA expressions, which may suggest that the resulting increase in bladder contractions reflect the muscarinic downstream involving higher intracellular calcium via inositol 1,4,5-trisphosphate (InsP3) and/or extracellular calcium influx to voltage-operated calcium channels (Andersson and Arner, 2004). Previous studies with isolated detrusor from both type 1 and type 2 diabetic mice showed that increased contractile response to muscarinic activation was linked to a higher expression and/or sensitization of the L-type channel coupling to M_3_ receptors to facilitate the entry of Ca^2+^ (Leiria et al., 2011, 2012). In addition, as we used the non-selective muscarinic agonist carbachol to evaluate cholinergic stimulated responses, we cannot rule out the involvement of nicotinic receptors in MGO-induced bladder dysfunction. Previous studies reported that nicotinic receptors are expressed in the mouse bladder mucosa (Zarghooni et al., 2007) and that its stimulation can alter the bladder reflex activity in rats (Kullman et al., 2008; Beckel and Birder, 2012). Furthermore, EFS-induced contractions, which reflect mainly the release of both acetylcholine and ATP from parasympathetic fibers, were higher in MGO compared with control group. Of interest, previous studies showed higher responses to EFS stimulation in both rat and mouse diabetic bladders (Tammela et al., 1994; Leiria et al., 2011). Moreover, the generalized depolarization by KCl induced similar contractile responses in both control and MGO groups, reinforcing that contractile machinery is not affected in MGO exposed bladders, but rather the responsiveness to cholinergic stimulation. However, further investigations are needed to understand the mechanisms by which MGO interacts with the cellular machinery of the urothelium and/or the detrusor. Additionally, no significant changes in the blood glucose levels were noted in MGO-exposed mice, indicating that overactive bladder induced by MGO takes place independently of hyperglycemia. Although several studies have attributed to chronic hyperglycemia a causal role in DBD development and progression (Daneshgari et al., 2017), some diabetic patients develop complications regardless of the glycemic control (Yudkin et al., 2010). Moreover, non-hyperglycemic obese patients may exhibit increased plasma MGO levels (Masania et al., 2016).

TRP channels are widely expressed in lower urinary tract tissues and play a critical role in the normal micturition reflex (for review: Andersson, 2019). TRPA1 is known to contribute to nociceptive responses evoked by endogenous and exogenous irritants (Oyama et al., 2017), including MGO itself (Bierhaus et al., 2012; Eberhardt et al., 2012). On the other hand, TRPV1 and TRPV4 are involved in bladder activity in response to mechanical or chemical stimulus (Andersson, 2019). We found that MGO induced a marked increase in TRPA1 expression, which may account for the bladder hyperreflexia. We also found that TRPV1 and TRPV4 are down-regulated in bladder tissues of MGO-exposed mice, which is indicative that that MGO impairs bladder pressure-sensory processes, culminating in the increased voiding frequency and detrusor instability. A previous urodynamic study in TRPV1 and TRPV4 knockout mice revealed that deletion of these receptors induced a dysfunctional micturition pattern, characterized by increases in voiding frequency and NVCs number (Yoshiyama et al., 2015).

Inflammation has been described as major contributor to the progressive organ damage in several diabetic complications, including DBD (Golbidi and Laher, 2010; Inouye et al., 2018). We found that MGO exposure led to evident inflammation of the bladder, characterized by tissue disorganization, edema of the lamina propria, partial loss of the urothelium and multiple eosinophil and lymphocyte infiltrates. Recently, MGO was shown to directly activate human macrophages (Bezold et al., 2019). Whether MGO can directly stimulate leukocyte to migrate remains to be determined. A previous study demonstrated that MGO promotes up-regulation of P-selectin, E-selectin and intercellular adhesion molecule-1 (ICAM-1), favoring the leukocyte influx in microvasculature (Su et al., 2012).

Methylglyoxal, as a powerful precursor of AGEs, has gained prominence as a key signaling molecule mediating diabetes pathophysiology(Allaman et al., 2015). Binding of AGEs to RAGE activates multiple signaling mechanisms, resulting in induction of NF-κB, amongst other factors (Akhand et al., 2001). In our study, MGO exposure did not change RAGE gene expression, but markedly increased its downstream NF-κB, a well-established transcription factor involved in the production of proinflammatory mediators, further propagating the inflammatory signal (Kierdorf and Fritz, 2013). Importantly, NF-κB is also a regulator of RAGE itself, representing a positive feedback mechanism of RAGE signaling, sustaining the inflammatory response (Kierdorf and Fritz, 2013). Furthermore, NF-κB is one of well-known iNOS inducers, an enzyme normally absent in the bladder, but largely expressed in inflammatory conditions (Austin et al., 2003), including patients with bladder pain syndrome/interstitial cystitis (BPS/IC; Logadottir et al., 2013). Our results showed that MGO markedly increased iNOS expression, which agrees with previous studies in chondrocytes *in vitro* (Huang et al., 2009). Once expressed, iNOS produces high levels of nitric oxide continuously that contributes to oxidative stress by increasing peroxynitrite (O_2_^–^) formation. Our data, however, showed no higher ROS levels in bladders of MGO-exposed mice. Moreover, a recent study demonstrated that ROS can modulate COX-2 expression and/or activity (Martínez-Revelles et al., 2013), a key pro-inflammatory enzyme responsible for prostaglandin synthesis in various tissues, including the bladder (Park et al., 1997). *In vitro* studies showed a significant increase of COX-2 expression in synovial cells incubated with MGO (Lin et al., 2016). However, our data showed no differences in COX-2 expression in whole bladder lysates of MGO compared with control groups, excluding this signaling pathway in the resulting bladder dysfunction.

Recently, numerous studies focused on targeting MGO therapeutically as an alternative for the management of diabetes complications. One very effective and safe MGO scavenger is the biguanide antihyperglycemic agent metformin, which can directly bind to the α-dicarbonyl group of MGO, thus preventing its ability to bind other proteins (Ruggiero-Lopez et al., 1999). Other studies also reported that metformin can increase the activity of the glyoxalase pathway, thereby increasing MGO detoxification (Beisswenger et al., 1999; Kender et al., 2014). Metformin reduces plasma MGO concentrations in type 2 diabetic patients (Kender et al., 2014), and is associated with amelioration of micturition symptoms in both diabetic patients (Brown et al., 2006) and high fat fed obese mice (Leiria et al., 2012). Therefore, further studies exploring the effects of metformin in voiding dysfunction by MGO are merited.

Amongst the limitations of this study, we evaluated the MGO effects only at a specific time of exposure. We recognize that further evaluation at multiple times points is necessary to better understand the consequences of chronic accumulation of MGO. In addition, due to the exogenous administration of MGO, it is unclear if the effects observed rely on elevated extracellular levels of MGO, rather than intracellular accumulation, as observed in diabetes. Thus, future studies will be needed to study in detail the effects of MGO to bladder function.

## Conclusion

Our research provided the first analysis of the deleterious effects of MGO on mouse bladder, which includes urodynamic changes and bladder inflammation that resemble DBD. Hence, MGO accumulation may be an important mediator for progressive structural and functional changes observed in DBD. Scavengers of MGO could be an option to ameliorate DBD. Further understanding of the molecular mechanisms underlying MGO effects may provide additional targets for therapeutic intervention.

## Data Availability Statement

All data generated for this study are included in the manuscript.

## Ethics Statement

All experimental protocols were carried out according to the Ethical Principles in Animal Research adopted by the Brazilian College for Animal Experimentation and were approved by the Institutional Committee for Ethics in Animal Research of the University of Campinas (Protocol no 4902-1).

## Author Contributions

MO, MM, and ET carried out the experiments. MO, FM, and EA analyzed the experimental results. MO and EA designed the experiments and wrote the manuscript.

## Conflict of Interest

The authors declare that the research was conducted in the absence of any commercial or financial relationships that could be construed as a potential conflict of interest. The handling Editor and reviewer PA declared their involvement as co-editors in the Research Topic, and confirm the absence of any other collaboration.
